# Bloch-like waves in random-walk potentials based on supersymmetry

**DOI:** 10.1038/ncomms9269

**Published:** 2015-09-16

**Authors:** Sunkyu Yu, Xianji Piao, Jiho Hong, Namkyoo Park

**Affiliations:** 1 Photonic Systems Laboratory, Department of Electrical and Computer Engineering, Seoul National University, Seoul 08826, Korea

## Abstract

Bloch's theorem was a major milestone that established the principle of bandgaps in crystals. Although it was once believed that bandgaps could form only under conditions of periodicity and long-range correlations for Bloch's theorem, this restriction was disproven by the discoveries of amorphous media and quasicrystals. While network and liquid models have been suggested for the interpretation of Bloch-like waves in disordered media, these approaches based on searching for random networks with bandgaps have failed in the deterministic creation of bandgaps. Here we reveal a deterministic pathway to bandgaps in random-walk potentials by applying the notion of supersymmetry to the wave equation. Inspired by isospectrality, we follow a methodology in contrast to previous methods: we transform order into disorder while preserving bandgaps. Our approach enables the formation of bandgaps in extremely disordered potentials analogous to Brownian motion, and also allows the tuning of correlations while maintaining identical bandgaps, thereby creating a family of potentials with ‘Bloch-like eigenstates'.

## Abstract (web summary)

Various approaches based on the iterative search have failed in the deterministic creation of bandgaps in random networks. Here Yu *et al*. reveal a deterministic pathway to bandgaps in random-walk potentials by applying the notion of supersymmetry to the wave equation.

## Graphical abstract

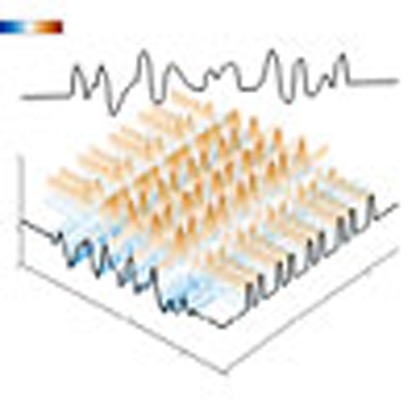

The isospectral problem posed via the question ‘Can one hear the shape of a drum?'^1^ introduced many fundamental issues regarding the nature of eigenvalues (sound) with respect to potentials (the shapes of drums). Following the demonstration presented in ref. 2, it was shown that it is not possible to hear the shape of a drum because of the existence of different drums (potentials) that produce identical sounds (eigenvalues), namely, isospectral potentials. Although the isospectral problem has deepened our understanding of eigenstates with respect to potentials and raised similar questions in other physical domains^3^, it has also resulted in various interesting applications such as the detection of quantum phases^4^ and the modelling of anyons^5^.

The field of supersymmetry^6^ (SUSY) shares various characteristics with the isospectral problem. SUSY, which describes the relationship between bosons and fermions, has been treated as a promising postulate in theoretical particle physics that may complete the standard model^6^. Although the experimental demonstration of this postulate has encountered serious difficulties and controversy, the concept of SUSY and its basis of elegant mathematical relations have given rise to remarkable opportunities in many other fields, for example, SUSY quantum mechanics^7^ and topological modes^8^. Recently, techniques from SUSY quantum mechanics have been utilized in the field of optics, thereby enabling novel applications in phase matching and isospectral scattering^9^,^10^,^11^,^12^, complex potentials with real spectra^13^ and complex Talbot imaging^14^.

In this paper, we propose a supersymmetric path for the generation of Bloch-like waves and bandgaps without the use of Bloch's theorem^15^. In contrast to approaches based on an iterative search for random networks^16^,^17^,^18^,^19^ with bandgaps, a deterministic route towards bandgap creation in the case of disordered potentials is achieved based on the fundamental wave equation. This result not only demonstrates that long-range correlation is a sufficient but not a necessary condition for Bloch-like waves^16^,^17^,^18^,^19^ but also enables the design of random-walk potentials with bandgaps. Such designs can facilitate the creation of a family of potentials with ‘Bloch-like eigenstates': identical bandgaps and tuneable long-range correlations, even extending to conditions of extreme disorder analogous to Brownian motion. We demonstrate that the counterintuitive phenomenon of ‘strongly correlated wave behaviours in weakly correlated potentials' originates from the ordered modulation of potentials based on spatial information regarding the ground state, which is the nature of SUSY. We also show that our approach for Bloch-like waves can be extended to multi-dimensional potentials under a certain condition, allowing highly anisotropic control of disorder.

## Results

### Relation between eigenstates and potential correlations

To employ the supersymmetric technique^7^,^9^, we investigate waves governed by the one-dimensional (1D) Schrodinger-like equation, which is applicable to a particle in nonrelativistic quantum mechanics or to a transverse electric mode in optics. Without a loss of generality, we adopt conventional optics notations for the eigenvalue equation *H*_o_*ψ*=*γψ*, where the Hamiltonian operator *H*_o_ is


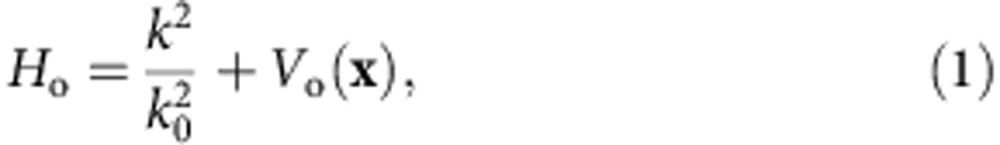


*k*=*i*∂_*x*_ is the wavevector operator, *k*_0_ is the free-space wavevector, *V*_o_(*x*)=–[*n*(*x*)]^2^ is the optical potential, *n*(*x*) is the refractive index profile, *ψ* is the transverse field profile and *n*_eff_ is the effective modal index for the eigenvalue 
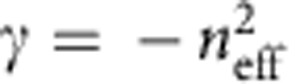
. Two independent methods are applied to equation (1) for verification, the Finite Difference Method^20^ (FDM) and the Fourier Grid Hamiltonian^21^ (FGH) method, whereby both yield identical results for the determination of bound states (see the Methods section).

**Figure 1:**
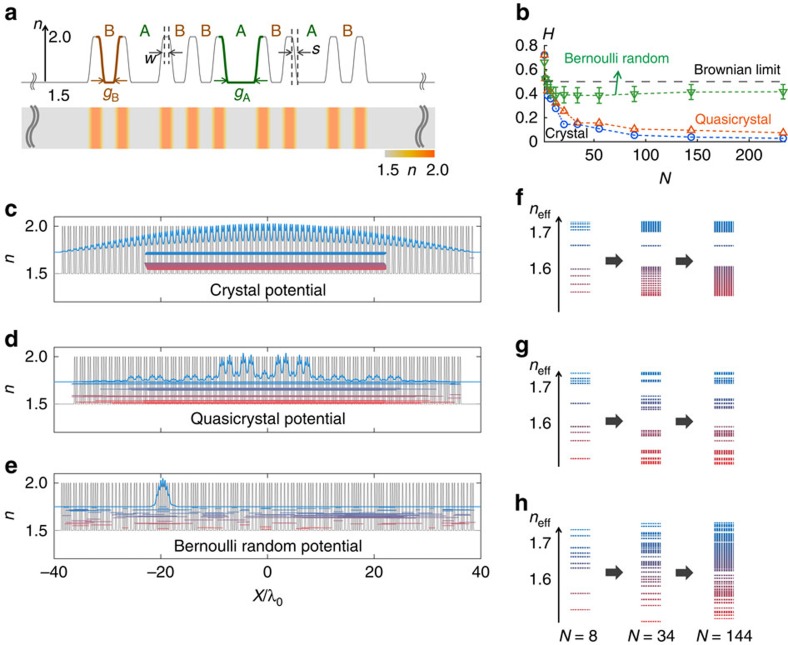
Relation between eigenstates and potential correlations. (**a**) Definitions of elements, illustrated for an example of a 1D Fibonacci quasicrystal (*N*=8). *g*_A_=600 nm, *g*_B_=200 nm, *w*=120 nm, *s*=140 nm and the wavelength is *λ*_0_=1,500 nm. (**b**) Hurst exponent *H* for each potential as a function of the sequence length *N*. The sequence lengths *N* are selected to be equal to those of Fibonacci quasicrystals. The *H* of the Bernoulli random potential is plotted with s.d. error bars for 200 statistical ensembles. The black dashed line represents the Hurst exponent of ideal Brownian motion (*H*=0.5). (**c**–**e**) Eigenstates of each potential. The blue curve represents the ground state of each potential, and the coloured lines represent the spectral (*n*_eff_) distributions of the eigenstates. (**f**–**h**) Evolutions of the band structures for different sequence lengths *N*: (**c**,**f**) for crystals, (**d**,**g**) for quasicrystals and (**e**,**h**) for Bernoulli random potentials. Note that the eigenstate inside the gap in **c** and **f** is a surface state for an even *N* (or an odd number of high-index regions) from the finite sizes of the potentials.

To examine the relationship between wave eigenspectra and the correlations of potentials, three types of random-walk potentials are analysed: crystals, quasicrystals and disordered potentials, which are generated by adjusting the refractive index profile. Figure 1a represents a 1D binary Fibonacci quasicrystal (the sixth generation with an inflation number, or sequence length, of *N*=8, substituting A→B and B→BA for each generation using A as the seed), where each element is defined by the gap between the high-index regions: A (or B) for a wider (or narrower) gap. The crystal and the disordered potential are generated using the same definition of elements, while the crystal has an alternating sequence (BABABA…), and the disordered potential has equal probabilities of A and B for each element (that is, it is a Bernoulli random sequence^22^ with probability *P*=0.5). To quantify the correlation, the Hurst exponent^23^,^24^ *H* is introduced (Fig. 1b; see the Methods section). As *N* increases, both the crystal and the quasicrystal have *H* values that approach 0 (that is, they exhibit ‘ballistic behaviour' with strong negative correlations^24^), in stark contrast to the Bernoulli random potential, which has *H*∼0.4 (close to ideal Brownian motion, with *H*=0.5).

Figure 1c–h illustrates the stationary eigenstates for each potential, which are calculated using the FDM and the FGH method. Consistent with previous studies^16^,^17^,^18^,^19^,^25^,^26^,^27^,^28^,^29^, Bloch-like waves with wide bandgaps are obtained for the ordered potentials of the crystal (Fig. 1c) and the quasicrystal (Fig. 1d), and the Bloch-like nature becomes more apparent with increasing *N* (Fig. 1f,g). By contrast, no bandgap is observed for the Bernoulli random potential, which lacks any correlations (Fig. 1e), especially for larger *N* (Fig. 1h); this lack of correlation originates from the broken coherence of this case, which hinders the destructive interference that is necessary for the formation of bandgaps. It should also be noted that many eigenstates are localized within this random potential, exhibiting a phenomenon that is widely known as Anderson localization^29^.

### Supersymmetric transformation for quasi-isospectral design

In light of the results in Fig. 1, we now consider the following question: ‘Is it possible to design nearly uncorrelated (or Brownian) potentials with *H*∼0.5 while preserving the original bandgaps?'. To answer this question positively, we exploit the SUSY transformation to achieve quasi-isospectral potentials^7^,^9^. In equation (1), it is possible to decompose the Hamiltonian operator as follows: *H*_o_–*γ*_0_=*NM*, where *N*=−*ik*/*k*_0_+*W*(*x*), *M*=*ik*/*k*_0_+*W*(*x*), *W*(*x*) is the superpotential that satisfies the Riccati equation *W*(*x*)^2^–*i*[*kW*(*x*)]/*k*_0_+*γ*_0_=*V*_o_(*x*) and *γ*_0_ is the ground-state eigenvalue of *H*_o_*ψ*_0_=*γ*_0_*ψ*_0_. Then, the inversion of the *N* and *M* operators yields the SUSY Hamiltonian *H*_s_ with the SUSY partner potential *V*_s_(*x*): *H*_s_=*MN*+*γ*_0_=*k*^2^/*k*_0_+*V*_s_(*x*). From the original equation *H*_o_*ψ*=*γψ*, the relation *H*_s_·(*Mψ*)=*γ*·(*Mψ*) is obtained, thus proving isospectrality with *γ* and the transformed eigenstates of *Mψ*^7^,^9^. For the later discussion of two-dimensional (2D) potentials, it is noted that the isospectrality between *H*_o_ and *H*_s_ can also be expressed in terms of the intertwining relation *MH*_o_=*H*_s_*M*=*MNM* (*MH*_o_*ψ*=*H*_s_(*Mψ*)=*γ*(*Mψ*) when *H*_o_*ψ*=*γψ*), where the operator *M* is the intertwining operator^30^,^31^.

The solution *W*(*x*) is simply obtained from the Riccati equation through *W*(*x*)=[∂_*x*_*ψ*_0_(*x*)]/[*k*_0_*ψ*_0_(*x*)] for unbroken SUSY^7^,^9^, which also provides the ground-state annihilation equation *Mψ*_0_=[*ik*/*k*_0_+*W*(*x*)]*ψ*_0_=*O*. Because *V*_s_(*x*)=–[*n*_s_(*x*)]^2^ is equivalent to


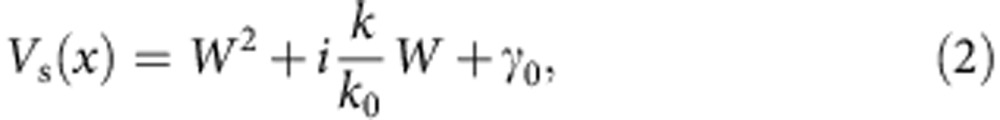


the index profile *n*_s_(*x*) after the SUSY transformation can finally be obtained as follows:


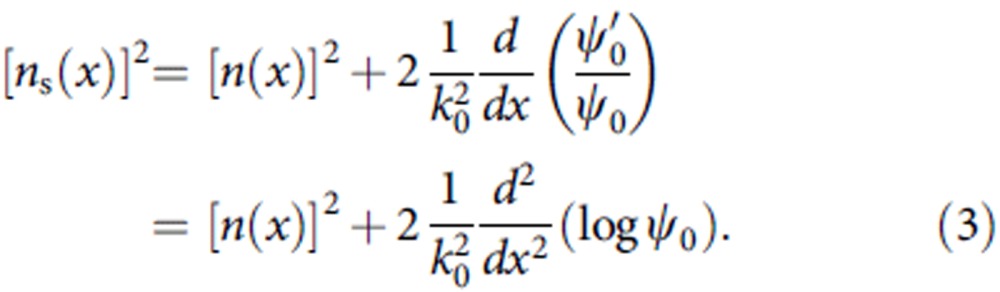


**Figure 2:**
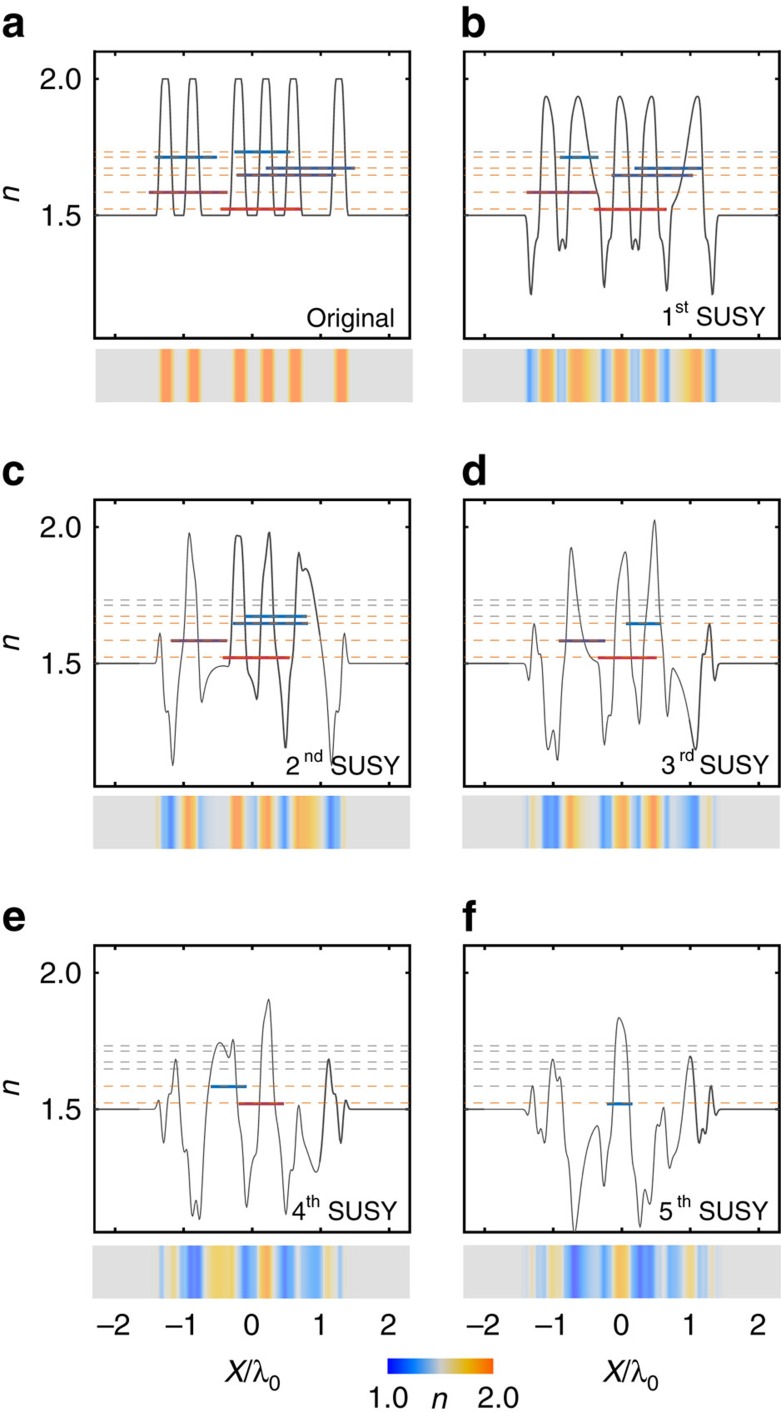
SUSY transformation for quasi-isospectral potentials. A 1D Fibonacci quasicrystal (*N*=5) is considered as an example. (**a**) Original potential. (**b**–**f**) 1st–5th SUSY-transformed potentials. The orange (or black) dotted lines represent the preserved (or annihilated) eigenstates. All eigenstates are calculated using both the FDM and FGH method, the results of which are in perfect agreement.

Equation (3) demonstrates that the SUSY transformation can be achieved deterministically based solely on the ground-state-dependent functionality. Figure 2 illustrates an example of serial SUSY transformations applied to the 1D Fibonacci quasicrystal potential defined in Fig. 1, where the small value of *N*=5 is selected for clarity of presentation. For each SUSY transformation, all eigenstates of each previous potential, except for the ground state, are preserved in the transformed spatial profiles, while the shape of the designed potential becomes ‘disordered' through ‘deterministic' SUSY transformations.

### Potentials with Bloch-like states and tuneable randomness

**Figure 3:**
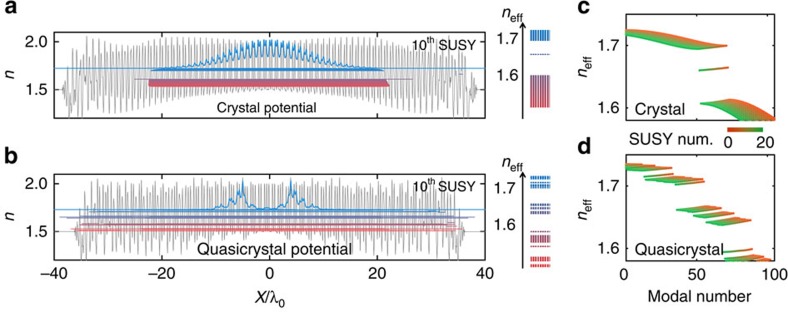
Eigenstates of SUSY-transformed crystals and quasicrystals. The 10th SUSY-transformed potentials and their eigenstates are depicted for (**a**) a crystal potential and (**b**) a quasicrystal potential. (**c**,**d**) The eigenvalues of the SUSY-transformed potentials as a function of the modal numbers of the crystal and quasicrystal potentials, respectively. The 0th SUSY-transformed potential corresponds to the original potential. *N*=144.

Because the presence of deterministic order is essential for Bloch-like waves and bandgaps, regardless of the presence of long-range correlations in their spatial profiles^16^,^17^,^18^,^19^,^25^,^26^,^27^,^28^,^29^, the ‘randomly-shaped' potentials (Fig. 2) that can be ‘deterministically' derived by applying SUSY transformations to ordered potentials offer the possibility of combining Bloch-like waves and disordered potentials. To investigate the wave behaviour associated with the SUSY transformation, we consider a larger-*N* regime in which the wave behaviours are clearly distinguished between ordered (Fig. 1f,g) and disordered potentials (Fig. 1h). Figure 3a,b presents the results obtained after the 10th SUSY transformation for the crystal (Fig. 3a,c) and the quasicrystal (Fig. 3b,d) with *N*=144. Although the shapes of the SUSY-transformed potentials and the spatial information of the eigenstates in Fig. 3a,b are markedly different from those of the corresponding original potentials in Fig. 1c,d, the eigenspectrum of each potential is preserved, save for the annihilation of the 10 lowest eigenstates, which is consistent with the nature of SUSY transformations. From the SUSY transformation *Mψ*={*ik*/*k*_0_+∂_*x*_*ψ*_0_(*x*)/[*k*_0_*ψ*_0_(*x*)]}·*ψ*, it is also expected that the distribution of the ground state *ψ*_0_(*x*) with respect to the original state *ψ* primarily affects the effective width^32^ of the transformed eigenstate *Mψ*. In crystals that have highly overlapped intensity profiles between eigenstates, the effective width of *ψ* decreases progressively from serial SUSY transformations due to the ‘bound' distribution of *ψ*_0_(*x*). For a quasicrystal, the variation of the effective width showed more complex behaviour owing to its spatially separated eigenstates (see Supplementary Note 1 and Supplementary Figs 1 and 2 for the comparison between crystal and quasicrystal potentials).

The eigenspectral conservation is apparent in Fig. 3c,d, which depicts the variation in the effective modal index that occurs during the SUSY transformations (up through the 20th SUSY transformation). As shown, the eigenspectrum of each potential is maintained from the original to the 20th SUSY transformation, save for a shift in the modal number, and, therefore, the bandgaps in the remainder of the spectrum are maintained during the serial SUSY transformations (∼125 states after the 20th SUSY transformation following the loss of the 20 annihilated states). Consequently, bandgaps and Bloch-like eigenstates similar to those of the original potentials are allowed in SUSY-transformed potentials with disordered shapes (Fig. 3a,b) that can be classified as neither crystals nor quasicrystals.

**Figure 4:**
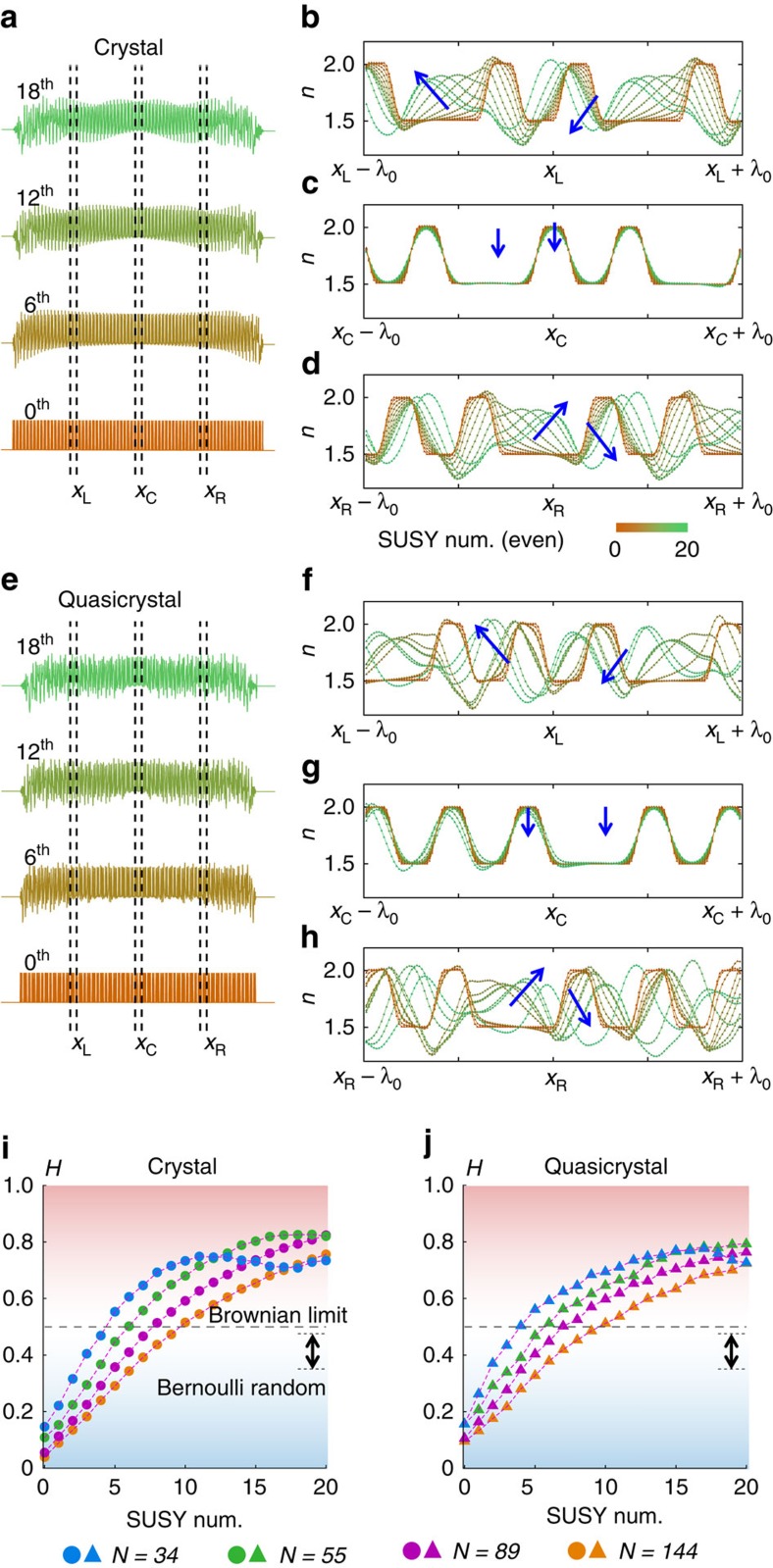
Correlation features of SUSY-transformed potentials. The evolutions of the potential profiles following the successive application of SUSY transformations (0th, 6th, 12th and 18th) for (**a**) a crystal and (**e**) a quasicrystal. (**b**–**d**,**f**–**h**) Magnified views (at *x*_L_, *x*_C_ and *x*_R_) of the potentials for even numbers of SUSY transformations (overlapped, up to the 20th transformation; the blue arrows indicate the direction of potential modulation). *N*=144 in **a**–**h**. Hurst exponents *H* as functions of the number of SUSY transformations are shown for (**i**) crystals and (**j**) quasicrystal potentials, with different sequence lengths (*N*=34, 59, 85 and 144). The red (or blue) region represents the regime of positive (or negative) correlation, whereas the white region corresponds to the uncorrelated Brownian limit. The arrow indicates the regime of the Bernoulli random potential (Fig. 1b).

Figure 4a–h illustrates the shape evolutions of the crystal and quasicrystal potentials that are induced through the SUSY process, demonstrating the increase in disorder for both potentials. Again, note that the SUSY-based modulation is determined by equation (3), starting from the ground-state profile *ψ*_0_(*x*), which is typically concentrated near the centre of the potential (Fig. 3a,b). To investigate the correlation features of SUSY-transformed potentials with bandgaps, we again consider the Hurst exponent. Figure 4i,j shows the Hurst exponents for the transformed crystal and quasicrystal potentials as functions of the number of SUSY transformations for different sequence lengths (*N*=34, 59, 85 and 144).

The figures show that, for successive applications of SUSY transformations, the Hurst exponents of the crystal and quasicrystal potentials (*H*=0∼0.1) increase and saturate at *H*∼0.8. For example, at *N*=144, the negative correlations (*H*<0.5) of the crystal and quasicrystal potentials (*H*=0∼0.1) become completely uncorrelated, with *H*=0.51 after the 10th SUSY transformation; the correlations are even weaker than that of the Bernoulli random potential (*H*=0.35∼0.48, Figs 1b and 4i,j) and approach the uncorrelated Brownian limit of *H*=0.5. After the 10th SUSY transformation, the correlation begins to increase again into the positive-correlation regime (*H*⩾0.5, with long-lasting, that is, persistent, potential shapes), thereby exhibiting a transition between negative and positive correlations in the potentials. This transition from an ‘anti-persistent' to ‘persistent' shape originates from the smoothing of the original potential caused by the slowly varying term 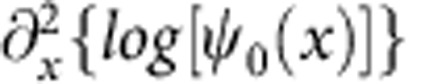 in equation (3), which is derived from the nodeless ground-state wavefunction *ψ*_0_(*x*).

We note that this *ψ*_0_(*x*)-dependent modulation shows a dependence on the size (or sequence length *N*) of the potentials; for a potential with a large size, *ψ*_0_(*x*) varies weakly over a wide range, thus decreasing the relative strength of the SUSY-induced modulation 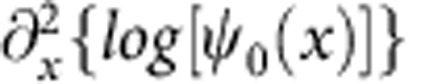 (Fig. 4i,j). Thereby, the number of SUSY transformations required for extreme randomness (*H*∼0.5) increases with the size of the potential (Fig. 4i,j, (*S*_B_, *N*)=(4, 34), (6, 55), (8, 89) and (10, 144), where *S*_B_ is the required SUSY transformations for *H*∼0.5). Eventually, the SUSY transformation to periodic potentials of infinite size *n*(*x*)=*n*(*x*+Λ) preserves the periodicity because the SUSY transformation with the Bloch ground state *ψ*_0_(*x*)=*ψ*_0_(*x+*Λ) will repeatedly result in periodic potentials *n*_s_(*x*)=*n*_s_(*x*+Λ).

These results reveal that the application of SUSY transformations to ordered (crystal or quasicrystal) potentials allows for remarkable control of the extent of the disorder while preserving Bloch-like waves and bandgaps. Therefore, a family of potentials with ‘Bloch-like eigenstates', for its members have identical bandgaps but tuneable disorders, can be constructed through the successive application of SUSY transformations to each ordered potential, with a range of disorder spanning almost the entire regime of Hurst exponents indicating negative and positive correlations (0≤*H*≤0.8), including the extremely uncorrelated Brownian limit of *H*∼0.5. As an extension, in Supplementary Note 2, we also provide the design strategy of random-walk discrete optical systems (composed of waveguides or resonators) that deliver Bloch-like bandgaps, starting from the first-order approximation of Maxwell's equations, that is, coupled mode theory^33^,^34^.

### Extension of SUSY transformations to 2D potentials

In stark contrast to the case of 1D potentials, which exclusively satisfy a 1:1 correspondence between their shape and ground state^7^, it is more challenging to achieve isospectrality in multi-dimensional potentials. Although studies have shown the vector-form SUSY decomposition of multi-dimensional Hamiltonians^35^,^36^,^37^, such an approach, which is analogous to the Moutard transformation^38^, cannot guarantee isospectrality. This approach only generates a pair of scalar Hamiltonians with eigenspectra that, in general, do not overlap but together compose the eigenspectrum of the other vector-form Hamiltonian^35^,^36^,^37^. Here we employ an alternative route^30^,^31^,^39^ starting from the intertwining relation *MH*_o_=*H*_s_*M* to implement a class of multi-dimensional isospectral potentials.

Without the loss of generality, we consider the 2D Schrodinger-like equations with the Hamiltonian of 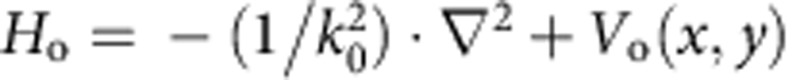 and its SUSY partner Hamiltonian 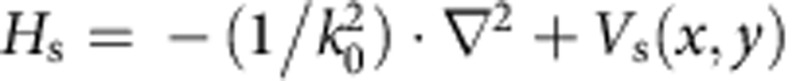. To satisfy the intertwining relation *MH*_o_=*H*_s_*M*, the ansatz for the intertwining operator *M* can be introduced^30^,^31^, similarly to the 1D case:


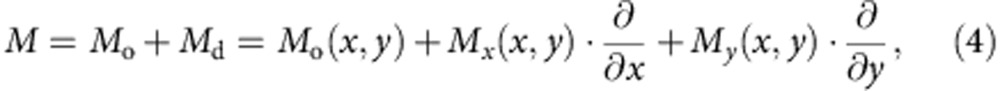


where *M*_o_, *M*_*x*_ and *M*_*y*_ are arbitrary functions of *x* and *y*. From equation (4), the intertwining relation *MH*_o_=*H*_s_*M* can be expressed in terms of operator commutators as follows:


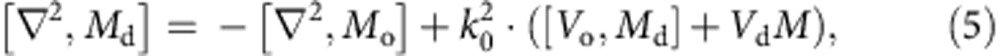


where *V*_d_ is the modification of the potential through the SUSY transformation: *V*_d_(*x*,*y*)=*V*_s_(*x*,*y*)–*V*_o_(*x*,*y*). Although here we focus on the 2D example, it is noted that equation (4) can be generalized to *N*-dimensional problems^30^,^31^ as *M*=*M*_o_(*x*_1_, *x*_2_, …, *x*_*N*_)+∑*M*_*i*_(*x*_1_, *x*_2_, …, *x*_*N*_)·*∂*_*i*_ while maintaining equation (5).

**Figure 5:**
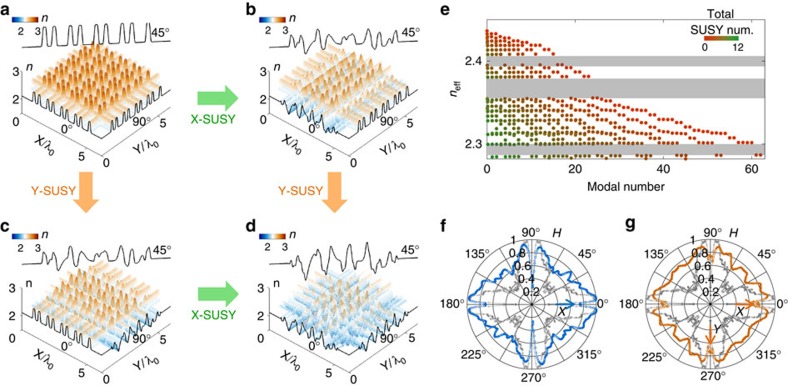
2D SUSY-transformed potentials with bandgaps maintained. The evolutions of the potential profiles following the application of SUSY transformations to the *x* and *y* axes are shown: (**a**) original and (**b**) *x* axis SUSY transformed (5th). (**c**) The *y* axis SUSY-transformed (5th), and (**d**) *x* and *y* symmetric SUSY-transformed (*x* axis: 5th; *y* axis: 5th) potentials. The spatial profiles of the potentials for 0° (*x* axis), 45° and 90° (*y* axis) are also overlaid in **a**–**d**. (**e**) The eigenvalues of the SUSY-transformed potentials as a function of the modal numbers. The grey regions denote bandgaps. The total SUSY number is the sum of the number of SUSY transformations for *x* and *y* axes (that is, 5 for both **b** and **c** and 10 for **d**). (**f**,**g**) The Hurst exponents for different directions of the 2D potentials that are (**f**) highly anisotropic (*x* axis: 5th, *y* axis: 0th) and (**g**) quasi-isotropic (*x* axis: 5th; *y* axis: 5th) SUSY transformations. The grey symbols in **f** and **g** are the Hurst exponents of the original potential.

The derivation in the Methods section (Equations 6, 7, 8, 9, 10, 11, 12, 13, 14, 15, 16, 17, 18, 19, 20, 21) starting from equation (5) demonstrates that the procedure of the 1D SUSY transformation can be applied to a 2D potential for each *x* and *y* axes independently, when the potential satisfies the condition of *V*_o_(*x*,*y*)=*V*_o*x*_(*x*)+*V*_o*y*_(*y*). We also note that serial 2D SUSY transformations are possible because the form of *V*_o_(*x*,*y*)=*V*_o*x*_(*x*)+*V*_o*y*_(*y*) is preserved during the transformation, consequently deriving a family of 2D quasi-isospectral potentials. Figure 5 shows an example of SUSY transformations in 2D potentials, maintaining Bloch-like eigenstates. Both the *x* and *y* axes cross-sections of the 2D original potential *V*_o_(*x*,*y*)=*V*_o*x*_(*x*)+*V*_o*y*_(*y*) have profiles of *N*=8 binary sequences (Fig. 5a), as defined in Fig. 1. Following the procedure of equations (17, 18, 19, 20, 21) in the Methods section, we apply SUSY transformations to the *x* and *y* axes separately, achieving the highly anisotropic shape of the potential as shown in Fig. 5b (the 5th *x* axis SUSY-transformed potential) and Fig. 5c (the 5th *y* axis SUSY-transformed potential). It is evident that this anisotropy can be controlled by changing the number of SUSY transformations for the *x* and *y* axes independently, and the isotropic application of SUSY transformations recovers the isotropic potential shape (Fig. 5d). Regardless of the number of SUSY transformations and their anisotropic implementations, the region of bandgaps of the original potential is always preserved (Fig. 5e). Interestingly, the annihilation by 2D SUSY transformation occurs not only in the ground state but also in all of the excited states sharing a common 1D ground-state profile (for details see the Methods section, Supplementary Note 3 and Supplementary Fig. 9). Consequently, the width of the bandgap can be slightly changed owing to the annihilation of some excited states near the bandgap.

To investigate the correlation features of 2D SUSY-transformed potentials, we quantify the angle-dependent degree of the correlation. Figure 5f,g shows the angle-dependent variation of the Hurst exponent for the anisotropic (the 5th *x* axis SUSY-transformed potential, Fig. 5b) and isotropic (the 5th *x* and *y* axes SUSY-transformed potential, Fig. 5d) disordered potential. Compared with the original potential (grey symbols in Fig. 5f,g, for Fig. 5a), *H* increases along the axis with the SUSY transformations (*x* axis in Fig. 5f and *x* and *y* axes in Fig. 5g). The potential is disordered at all angles, especially in the diagonal directions (±45°), owing to the projection of the SUSY-induced disordered potential shapes (45° profiles in Fig. 5b–d).

## Discussion

To summarize, by employing supersymmetric transformations, we revealed a new path toward the deterministic creation of random-walk potentials with ‘crystal-like' wave behaviours and tuneable spatial correlations, extending the frontier of disorder for Bloch-like waves and identical bandgaps. Despite their weak correlations and disordered shapes, SUSY-transformed potentials retain the deterministic ‘eigenstate-dependent order' that is the origin of bandgaps, which is in contrast to the hyperuniform^18^,^40^,^41^,^42^ disorder of pointwise networks and deterministic aperiodic structures such as quasicrystals^28^,^43^ or the Thue–Morse^44^ and Rudin–Shapiro^45^ sequences. We also extend our discussion to multi-dimensions, achieving highly anisotropic or quasi-isotropic disordered 2D potentials, while preserving bandgaps. Our results, which were obtained based on a Schrodinger-like equation, reveal a novel class of Bloch-wave disorder that approaches the theoretical limit of Brownian motion while maintaining wide bandgaps identical to those of existing crystals or quasicrystals in both electronics and optics. We further envisage a novel supersymmetric relation, based on the famous SUSY theory in particle physics, between ordered potentials and disordered potentials with coherent wave behaviours in solid-state physics. The extension of the SUSY transformation to non-Schrodinger equations, for example, transverse magnetic modes in electromagnetics (as investigated in the supplementary material of ref. 11), or to the approximated Hamiltonians applicable to arbitrary-polarized optical elements (Supplementary Note 2) will be of importance for future applications, for example, polarization-independent bandgaps based on dual-polarized eigenstates^46^.

## Methods

### Details of the FDM and FGH method

The FDM utilizes the approximation of the second-derivative operator in the discrete form^20^, and the FGH method, as a spectral method, uses a planewave basis with operator-based expressions in a spatial domain^21^. In both methods, the Hamiltonian matrices are Hermitian because of the real-valued potentials, thus enabling the use of Cholesky decomposition to solve the eigenvalue problem. To ensure an accurate SUSY process, Rayleigh quotient iteration is also applied to obtain the ground-state wavefunction. The boundary effect is minimized through the use of a buffer region (*n*=1.5) of sufficient length (30 μm=20*λ*_0_) on each side. Deep subwavelength grids (Δ=20 nm=*λ*_0_/75) are also used for the discretization of both the 1D and 2D potentials.

### Calculation of the Hurst exponent

First, the discretized refractive index *n*_*p*_ (*p*=1, 2, …, *N*) is obtained at *x*_*p*_=*x*_left_+(*p*−1)·Δ, where *x*_left_ is the left boundary of the potential, which is of length *L*=(*N*−1)·Δ. Partial sequences *X*_*q*_ of *n*_*p*_ for different length scales *d* are then defined (2≤*d*≤*N* and 1≤*q*≤*d*). For the mean-adjusted sequence *Y*_*q*_=*X*_*q*_–*m*, where *m* is the mean of *X*_*q*_, we define the cumulative deviate series *Z*_*r*_ as


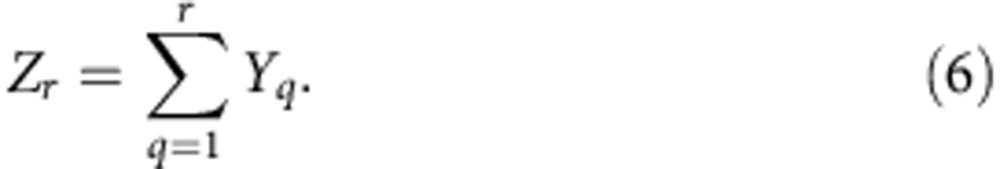


The range of cumulative deviation is defined as *R*(*d*)=max(*Z*_1_, *Z*_2_, …, *Z*_*d*_)–min(*Z*_1_, *Z*_2_, …, *Z*_*d*_). Using the s.d. *S*(*d*) of *Y*_*q*_, we can now apply the power law to the rescaled range *R*(*d*)/*S*(*d*) as follows:


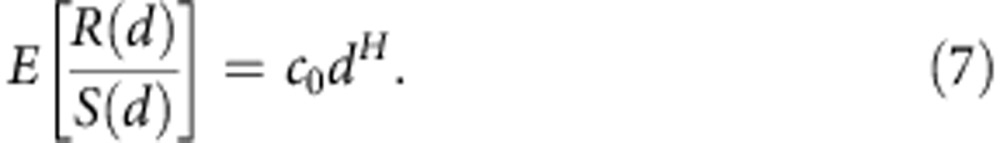


This yields log(*E*[*R*(*d*)/*S*(*d*)])=*H*·log(*d*)+*c*_1_, where *E* is the expectation value and *c*_0_ and *c*_1_ are constants. *H* is then obtained through linear polynomial fitting: *H*=0.5 for Brownian motion, 0≤*H*<0.5 for long-term negative correlations with switching behaviours, and 0.5<*H*≤1 for long-term positive correlations such that the sign of the signal is persistent.

### The condition for 2D isospectral potentials

By assigning *M*=*M*_o_+*M*_d_ (Equation 4) to the intertwining relation *MH*_o_=*H*_s_*M* with explicit forms of *H*_o_ and *H*_s_, it becomes 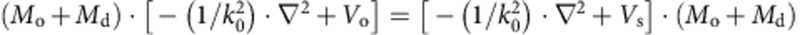. Thus, we obtain equation (5); 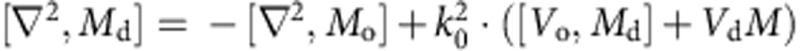, where *V*_d_=*V*_s_–*V*_o_. Each commutator in equation (5) is also expressed as


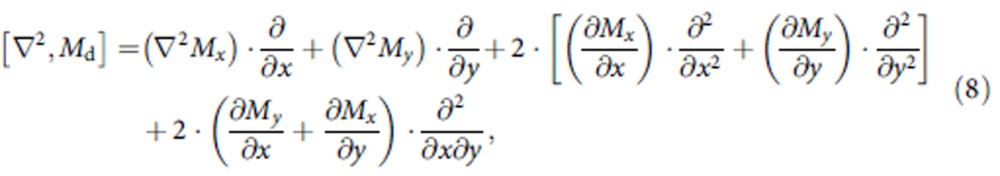



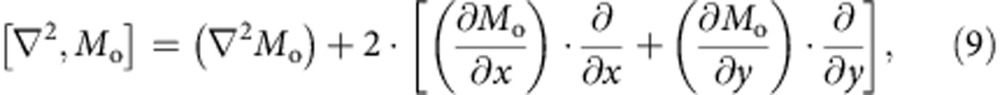



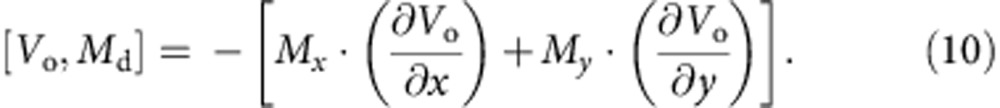


It is noted that the higher-order (⩾2) derivatives in equation (5) originate from the third and fourth terms in the right-hand side of equation (8). Comparing equation (8) with equations (9, 10), all of the higher-order derivatives should be removed to satisfy equation (5). This then directly leads to the preconditions *M*_*x*_ and *M*_*y*_; ∂_*x*_*M*_*x*_=0, ∂_*y*_*M*_*y*_=0 and ∂_*x*_*M*_*y*_+∂_*y*_*M*_*x*_=0, which hold only for *M*_*x*_(*x*,*y*)=*M*_*x*_(*y*)=*a*_*x*_–*by* and *M*_*y*_(*x*,*y*)=*M*_*y*_(*x*)=*a*_*y*_+*bx* where *a*_*x*_, *a*_y_ and *b* are arbitrary constants.

By applying *M*_*x*_(*y*), *M*_*y*_(*x*), and equations (8, 9, 10) to equation (5), we achieve two linear and one nonlinear equations for three unknowns *M*_o_, *V*_o_ and *V*_d_ as


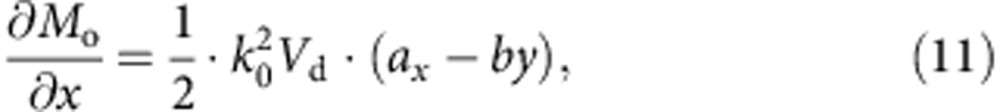



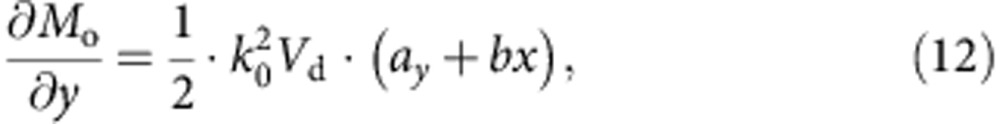



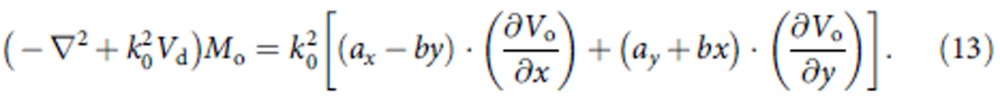


As a particular solution, we consider the case of *b*=0 for simplicity. In this case, from equations (11, 12), *M*_o_ and *V*_d_ are determined in the form of *M*_o_=*f*(*ρ*) and *V*_d_=*∂*_*ρ*_*f*(*ρ*), where *ρ*=*k*_0_^2^·(*a*_*x*_*x*+*a*_*y*_*y*)/2 is the transformed coordinate and *f* is an arbitrary function of *ρ*. By substituting *M*_o_ and *V*_d_, equation (13) then becomes


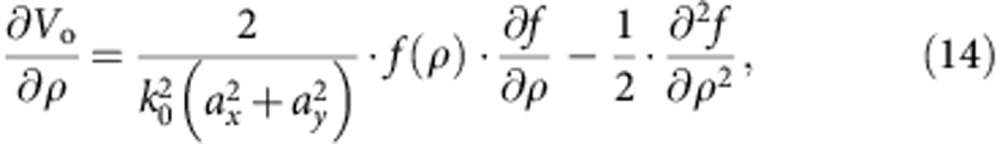


which reveals the proper form of the 2D potential *V*_o_ for SUSY transformations


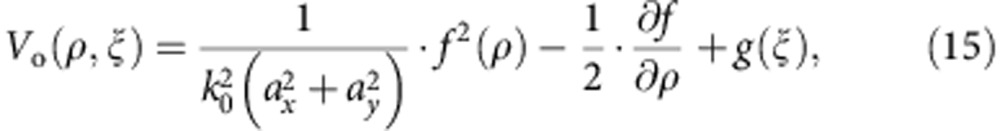


where *ξ*=*k*_0_^2^·(*c*_*x*_*x*+*c*_*y*_*y*)/2 is the transformed coordinate perpendicular to *ρ*, with *a*_*x*_·*c*_*x*_+*a*_*y*_·*c*_*y*_=0. The supersymmetric potential *V*_s_=*V*_o_+*V*_d_ then becomes


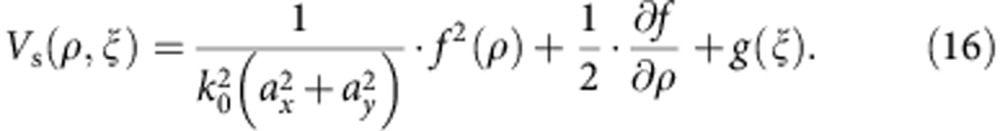


Using equations (15, 16), now we can implement the procedure of serial 2D SUSY transformations. First, because of equation (15), *V*_o_ should have the form of *V*_o_(*ρ*,*ξ*)=*V*_*oρ*_(*ρ*)+*V*_*oξ*_(*ξ*) for two Cartesian axes of *ρ* and *ξ*. In this case, the corresponding *f*(*ρ*) is obtained by solving the following Riccati equation:


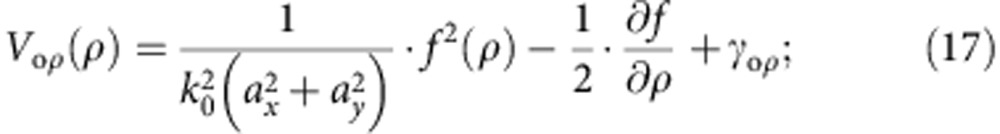


Its particular solution is listed as 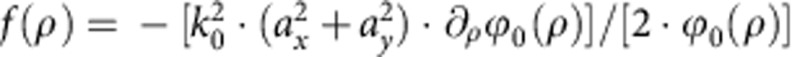 (ref. 47); where *ϕ*_0_(*ρ*) is the nodeless ground state with the eigenvalue *γ*_o*ρ*_ in the corresponding 1D Schrodinger-like equation


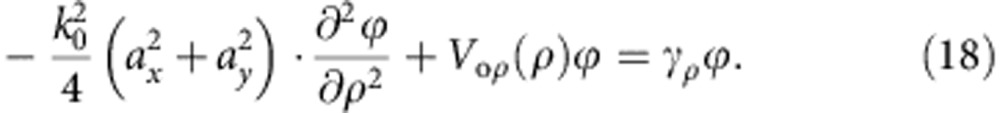


With the obtained *f*(*ρ*), we finally achieve the SUSY-transformed potential along the *ρ*-axis satisfying the isospectrality, *V*_s_(*ρ*,*ξ*)=*V*_o_(*ρ*,*ξ*)+∂_*ρ*_*f*(*ρ*), or


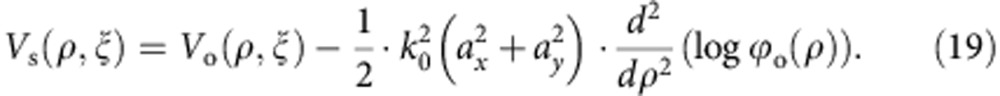


Equivalently, the SUSY transformation along the *ξ* axis is


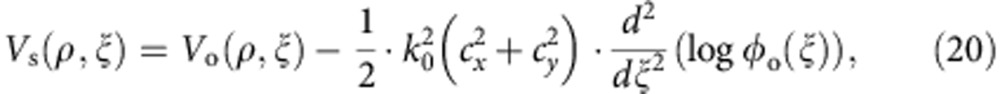


where *φ*_0_(*ξ*) is the nodeless ground state with the eigenvalue *γ*_oξ_ in the following equation:


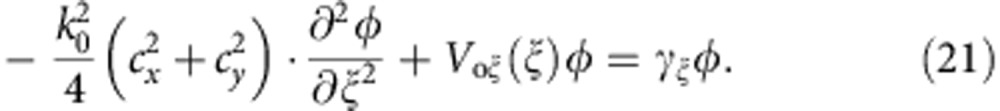


Note that, after the SUSY transformation for the *ρ* or *ξ* axes, *V*_s_(*ρ*,*ξ*) still preserves the form of *V*_s_(*ρ*,*ξ*)=*V*_*sρ*_(*ρ*)+*V*_*sξ*_(*ξ*) (Equations 19, 20), which is the necessary condition for the SUSY transformation of 2D potentials. Therefore, serial SUSY transformations can be applied to 2D arbitrary potentials of the form *V*_o_(*ρ*,*ξ*)=*V*_*oρ*_(*ρ*)+*V*_*oξ*_(*ξ*), and the level of SUSY transformations can be controlled independently for each axis, allowing highly anisotropic potential profiles. In addition, by assigning nonzero *b*, the allowed potential of *V*_o_(*x*,*y*) can be extended to non-separated forms^30^,^31^.

### The eigenstate annihilation in 2D SUSY transformations

In stark contrast to the ground-state annihilation in 1D SUSY transformations, the annihilation by 2D SUSY transformations is not restricted to the ground state. For simplicity, consider the case of 
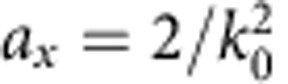
 and *a*_*y*_=0 for *ρ*=*x* and *ξ*=*y* without any loss of generality. The Hamiltonian, which can be SUSY transformed, is then expressed as 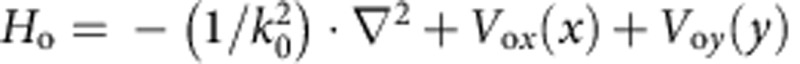 for the eigenvalue equation *H*_o_*ψ*=*γψ*. For the *x* axis SUSY transformation, the following equation should be satisfied to annihilate the SUSY-transformed eigenstate because *Mψ*=*O*:


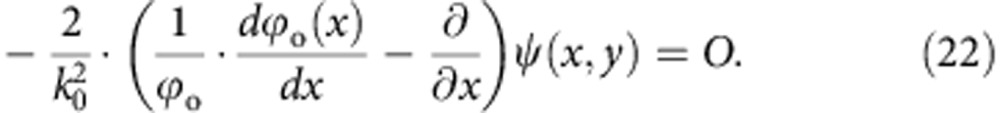


Note that equation (22) is satisfied when *ψ*(*x*,*y*)=*ϕ*_0_(*x*)·*φ*(*y*), allowing the separation of variables in the 2D eigenvalue equation *H*_o_*ψ*=*γψ* as


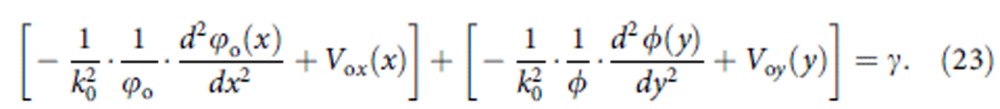


It is noted that the first brace has the fixed constant of *γ*_o*x*_, the ground-state eigenvalue of the 1D Schrodinger-like equation with the potential *V*_o*x*_(*x*). Meanwhile, because the second brace can be any eigenvalues of the solution *φ*(*y*) in the 1D Schrodinger-like equation with the potential *V*_o*y*_(*y*), it is clear that the annihilation by 2D SUSY transformation occurs not only in the ground state but also in all of the excited states sharing *ϕ*_0_(*x*). The detailed illustration of this result is shown in Supplementary Note 3 and Supplementary Fig. 9.

## Additional information

**How to cite this article:** Yu, S. *et al*. Bloch-like waves in random-walk potentials based on supersymmetry. *Nat. Commun.* 6:8269 doi: 10.1038/ncomms9269 (2015).

## Supplementary Material

Supplementary Information Supplementary Figures 1-9, Supplementary Note 1-3 and Supplementary References
