# Supplementary material for: Bloch-like waves in random-walk potentials based on supersymmetry
Source: Nat Commun. 2015 Sep 16;6:8269. doi: 10.1038/ncomms9269 (PMC4595658; doi:10.1038/ncomms9269)
Supplement: Supplementary Information — Supplementary Figures 1-9, Supplementary Note 1-3 and Supplementary References [file ncomms9269-s1.pdf]

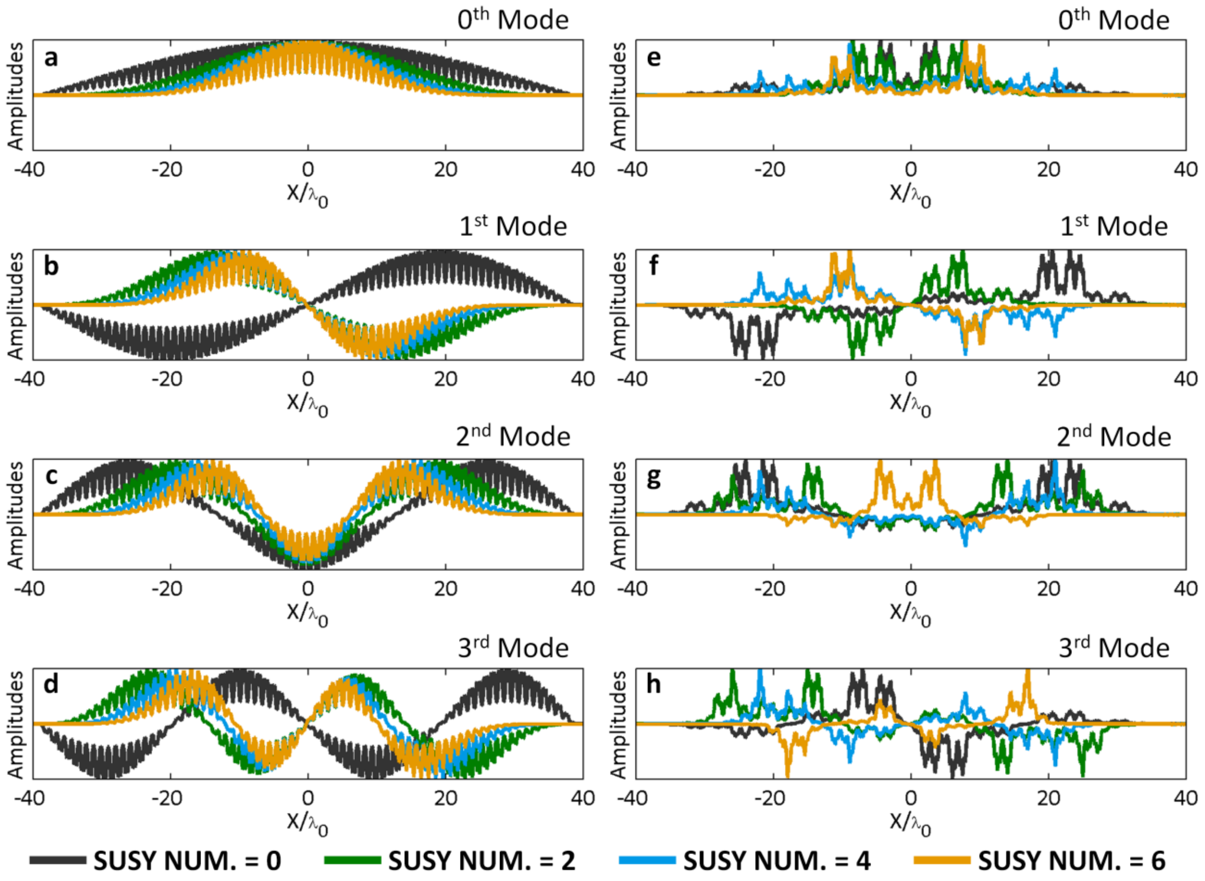

Supplementary Figure 1. The spatial variation of eigenstates from SUSY transformations (black: 0, green: 2, blue: 4, orange: 6 SUSY transformations): crystals (**a-d**) and quasicrystals (**e-f**) for different mode numbers (**a,e**,: 0th, **b,f**,: 1st, **c,g**,: 2nd, **d,h**,: 3rd mode). N = 144 and the initial shapes of potentials are same as those used in the main manuscript.

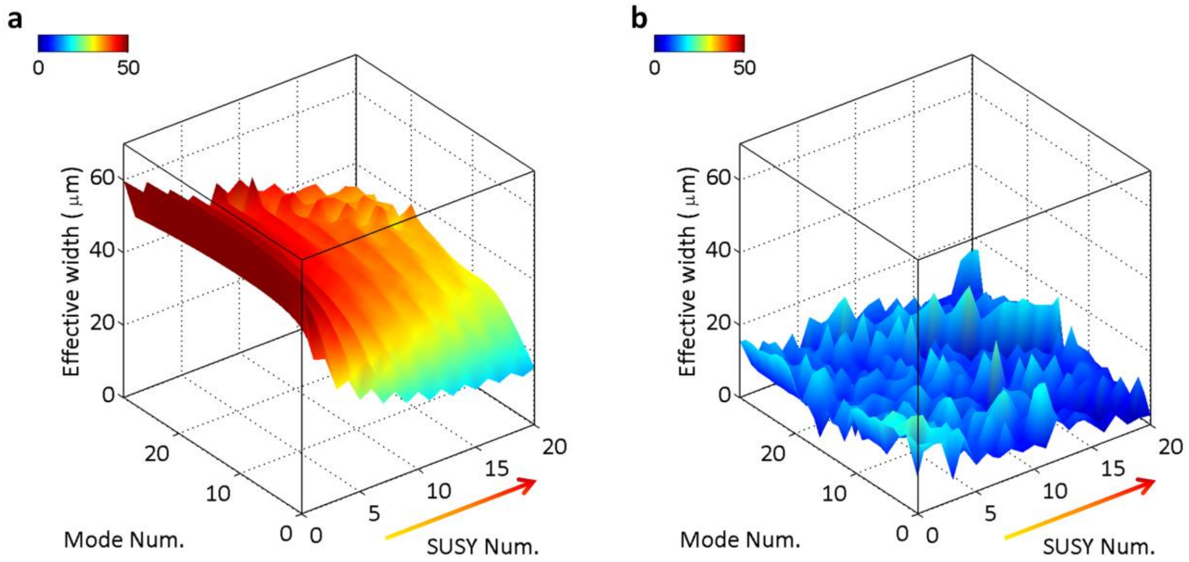

Supplementary Figure 2. The variation of the effective widths of the eigenstates from SUSY transformations: a, in the crystal, and **b,** in the quasicrystal. The number of SUSY transformations changes from 0 to 20. N = 144 and the initial shapes of potentials are same as those used in the main manuscript.

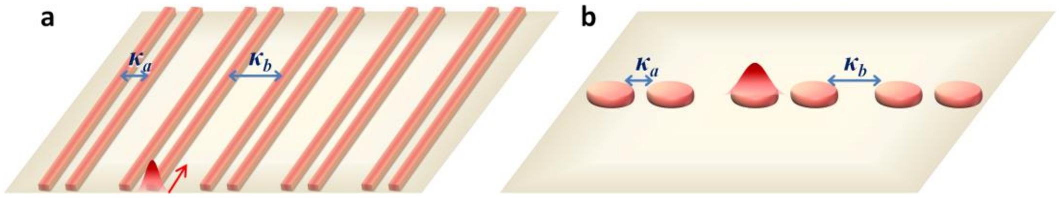

Supplementary Figure 3. Binary photonic molecules a, A waveguide-based photonic molecule of the spatial CMT ($\xi = x$). **b,** A resonator-based photonic molecule of the temporal CMT ($\xi = t$).

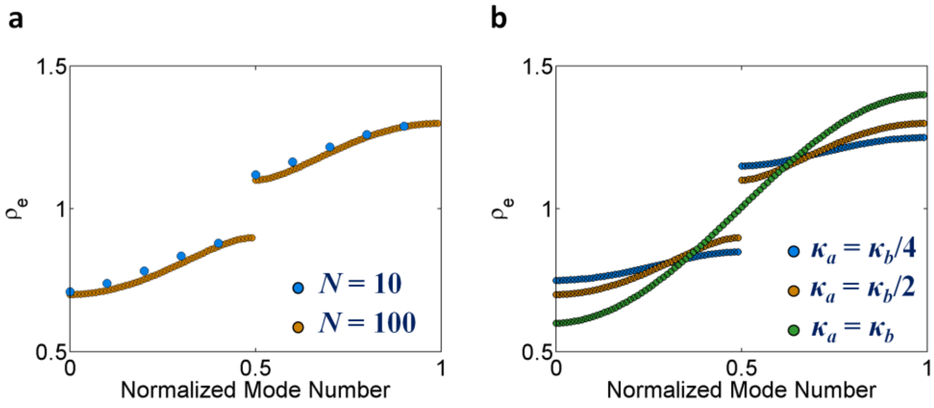

Supplementary Figure 4. Eigenspectra of binary photonic molecules for **a,** different N (κ_a = 0.1) and **b,** different κ_a (N = 100). ρ_0 = 1 and κ_b = 0.2 for all cases.

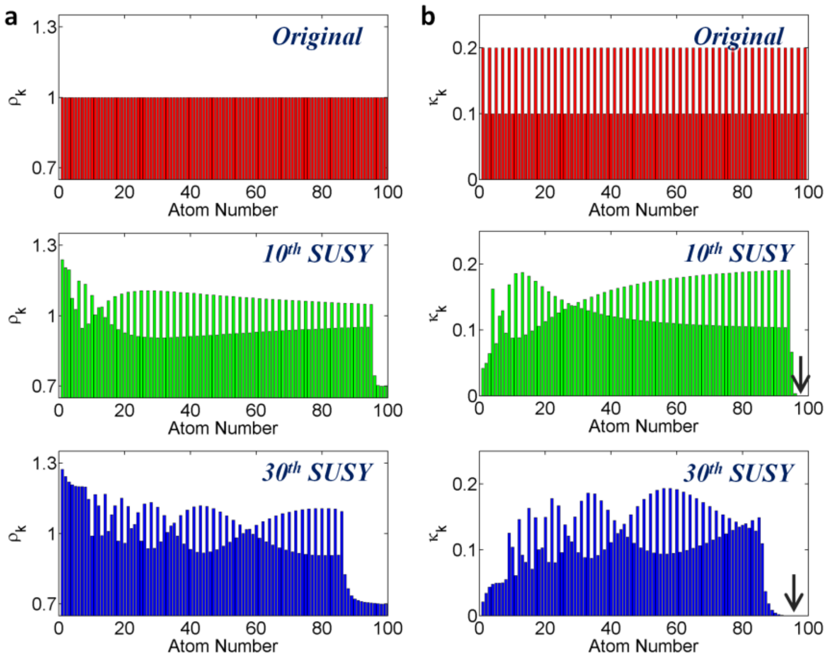

Supplementary Figure 5. The variation of the CMT parameters from the SUSY transformations a, The self-evolution for each atom and **b,** the coupling between atoms for the original, 10th, and 30th SUSY-transformed photonic molecules. The original binary molecule has the following parameters: κ_a = 0.1, κ_b = 0.2, ρ_0 = 1 and N = 100. Black arrows denote the decoupling.

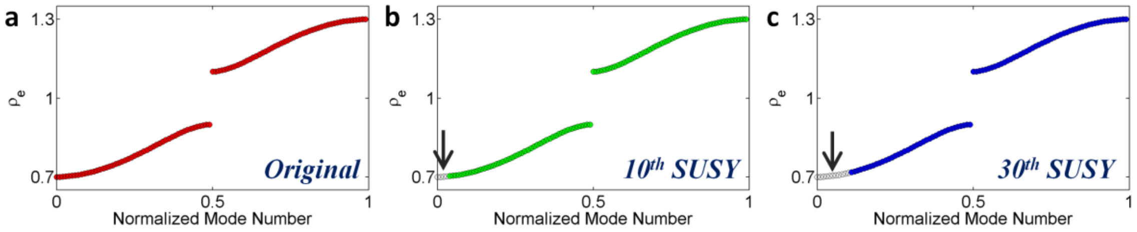

Supplementary Figure 6. Eigenspectra of SUSY-transformed photonic molecules in Supplementary Fig. 3 a, Original binary photonic molecule. **b,** The 10th and **c,** 30th SUSY-transformed photonic molecules. Black arrows denote the eigenstates of the decoupled atoms.

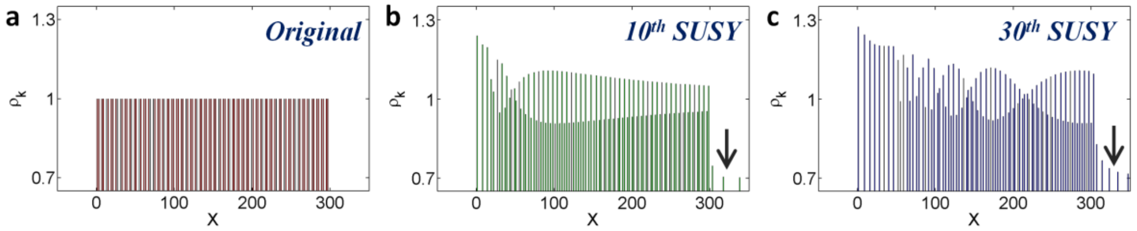

Supplementary Figure 7. Spatial distribution of optical potentials for **a,** an original binary photonic molecule, **b,** the 10th and **c,** 30th SUSY-transformed photonic molecules (corresponding to CMT parameters used in Supplementary Fig. 5). The black arrows denote the positions of decoupled atoms. To convert the coupling coefficients to the physical locations of each atom, the coupling coefficients are defined by setting two conditions: κ = 0.1 for Δx = 3 and κ = 0.2 for Δx = 1.

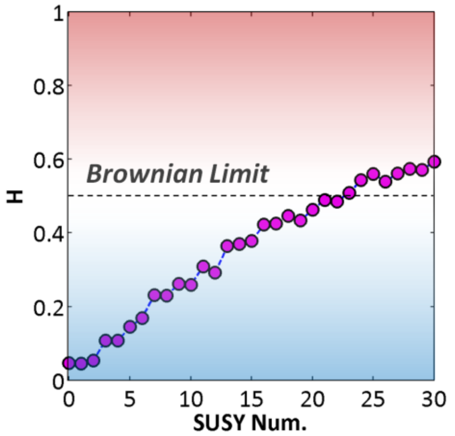

Supplementary Figure 8. Hurst exponents *H* for each transformed photonic molecule as a function of the number of SUSY transformations. The red (or blue) region represents the regime of positive (or negative) correlation, whereas the white region corresponds to the uncorrelated Brownian limit.

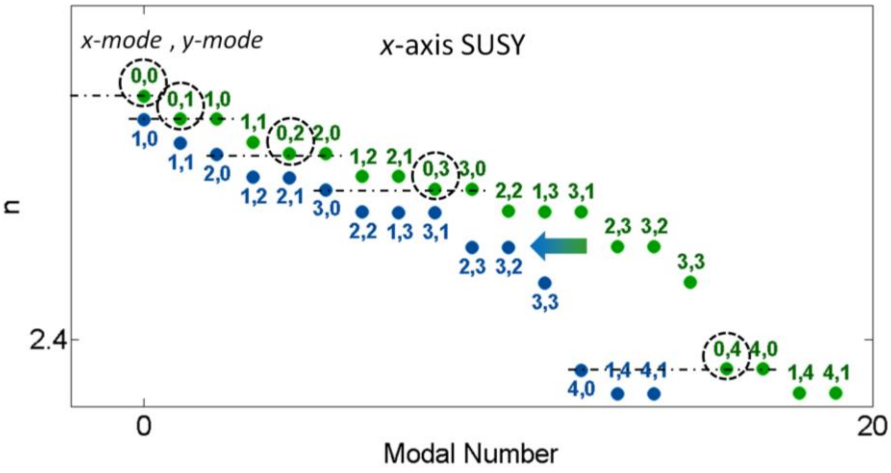

Supplementary Figure 9. The annihilation of the eigenstates from the *x*-axis SUSY transformation in 2D potentials. Out of various original eigenstates (green symbols), only the eigenstates with the 0th mode profile along the *x*-axis (black dotted circles) are annihilated. The blue symbols are SUSY-transformed eigenstates. The initial shapes of the potentials are the same as those used in Fig. 5 of the main manuscript.

Supplementary Note 1. The localization based on SUSY transformations

Supplementary Figure 1 shows examples of SUSY-transformed eigenstates (from the 0th to the 3rd modes, $M\psi = \{ik / k_0 + \partial_x\psi_0(x) / [k_0\psi_0(x)]\}\cdot\psi)$ in crystals and quasicrystals. In the case of the crystal with eigenstates that have highly overlapping intensity profiles, the 'bound' profile of $\psi_0(x)$ decreases the spatial bandwidth of each eigenstate with the contribution of $\{\partial_x\psi_0(x) / [k_0\psi_0(x)]\}\cdot\psi$. However, because the eigenstates in the quasicrystal are already spatially separated, in contrast to those in the crystal, the spatial modification by $\psi_0(x)$ occurs in a much more complex manner (Supplementary Figs 1e-1h).

To quantify the localization of eigenstates in SUSY-transformed potentials, we introduce the definition of the effective width[1] w_{eff} based on the inverse participation ratio, as

$$w_{\mathrm{eff}} = \frac{\left[\int I(x)dx\right]^2}{\int I^2(x)dx}, \tag{1}$$

where $I(x)$ is the intensity of each eigenstate. Supplementary Figure 2 shows the change of effective width for the 30 lowest eigenvalue modes by serial SUSY transformations (arrows in Supplementary Fig. 2). As shown, the effective width decreases gradually in the crystal potentials, whereas no tendency is observed in the quasicrystal potentials. However, for both crystals and quasicrystals, the cases of localized eigenstates were found from serial SUSY transformations.

Supplementary Note 2. Bloch-like waves in discrete optical systems based on coupled mode theory

The notion of discrete optical systems has played a critical role in the design of optical devices. By interpreting the actual landscape of optical potentials (permittivity and permeability) as a network of optical elements (or 'photonic molecule'[2,3] composed of 'photonic atoms'), the flow of light can be understood through simplified calculations without the need to solve Maxwell's equations directly. Classically, guided-wave platforms have been considered as the network composed of waveguides and resonators[4], and photonic crystals of various symmetries have been treated as tightly bound networks of dielectric atoms[5]. Recently, metamaterials have been investigated in the context of the interplay between electric and magnetic dipoles with elementary oscillations[6,7], *e.g.*, the Lorentz model.

Coupled mode theory (CMT)[4] is a powerful technique for investigating discrete optical systems based on the 1st-order approximation of wave equations, not only for the description of spatial beam dynamics in guided-wave platforms[8] but also for the analysis of coupled resonances[9] or metamaterials[10] in the temporal domain. Due to its simplified and generalized formulation with well-matched results, CMT have also been applied to the investigation of Bloch optical potentials, as shown in the studies of waveguide grating[11], Bloch oscillation[12], and the slow light structure based on coupled-resonator optical waveguides (CROW)[13].

Extending the discussion of the main manuscript, in Supplementary Note 2, we derive Bloch-like wave families based on supersymmetry (SUSY) in 'discrete' optical systems by applying CMT to the notion of photonic molecules[2,3] with periodicity.

Hamiltonian equation of photonic molecules based on CMT

In a 1-dimensional (or 'nearest-neighbor' coupling) problem, the elementary equation of CMT for the kth photonic atom is as follows:

$$\frac{d}{d\xi}\psi_k = -i\rho_k\psi_k + i\kappa_{k,k-1}\psi_{k-1} + i\kappa_{k,k+1}\psi_{k+1}, \qquad (2)$$

where ξ is a spatial axis x (or a time axis t) in the spatial (or temporal) CMT, ψ_k and ρ_k are the field amplitude and the self-evolution term of the kth atom, respectively (ρ_k is a wavevector in a spatial CMT or a resonant frequency in a temporal CMT), and κ is the coupling coefficient between atoms. The governing equation of a photonic molecule, composed of N photonic atoms, can then be expressed as the eigenvalue equation $H_o\Psi_e = \rho_e\Psi_e$ where ρ_e is an eigenvalue, Ψ_e is a corresponding eigenvector, and H_o is a Hamiltonian matrix in a tridiagonal form,

$$H_o = \begin{bmatrix} \rho_1 & -\kappa_{12} & & & \\ -\kappa_{21} & \rho_2 & -\kappa_{23} & & \\ & -\kappa_{32} & \rho_3 & \ddots & \\ & & \ddots & \ddots & -\kappa_{N-1,N} \\ & & & -\kappa_{N,N-1} & \rho_N \end{bmatrix}. \qquad (3)$$

If the photonic molecule is Hermitian without magneto-optical effects, the coupling between photonic atoms is symmetric[4] as $\kappa_{ij} = \kappa_{ji}$.

Band analysis of crystalline photonic molecules

Consider the case of 1-dimensional crystalline photonic molecules satisfying the Hermiticity ($\kappa_{ij} = \kappa_{ji}$, $\kappa_{k,k+1} = \kappa_k$). Here, we investigate the binary atomic distribution in the same way as in the main manuscript (CMT parameters of identical $\rho_k = \rho_0$ for all k, and $\kappa_{2m+1} = \kappa_a$, $\kappa_{2m+2} = \kappa_b$ for $m = 1, 2, \ldots$). Supplementary Figure 3 shows the schematics of binary photonic molecules, each for the waveguide-based example with a spatial CMT model[8] and the resonator-based example with a temporal CMT model[9].

Supplementary Figure 4 shows CMT-calculated eigenspectra as a function of modal number for the discrete system shown in Supplementary Fig. 3. Due to the binary

arrangement, a bandgap is achieved around the self-evolution term ρ_0 = 1. Note that the construction of the bandgap can be understood in terms of the repulsion (determined by κ_a) of even and odd parity-modal bands (formed by κ_b)[14]. By increasing the number of photonic atoms N, the eigenspectrum approaches the continuous band with a well-known cosine form[13,14], which is the feature of the lattice having *periodic* couplings (Supplementary Fig. 4a). The width of the bandgap is determined by the contrast between κ_a and κ_b, which is the same as other photonic crystals (Supplementary Fig. 4b).

SUSY-based random-walk photonic molecules: CMT modelling

To achieve the SUSY transformation for the CMT-based Hamiltonian equation $H_o\Psi_e = \rho_e\Psi_e$ with the process of the ground-state (ρ_{e0}) annihilation, the modified matrix $H_o' = H_o - \rho_{e0}I$ should be decomposed as $H_o' = M^\dagger M$, which is identical to the main manuscript. Due to the Hermitian and positive-definite features of H_o', we apply the Cholesky decomposition as demonstrated in the phase-matching design based on SUSY transformations (refs 15-17), which gives the upper triangular matrix M. The SUSY-transformed Hamiltonian H_s is then defined as $H_s = MM^\dagger + \rho_{e0}I$. Note that because H_o' is a tridiagonal matrix, $MM^\dagger$ and thus H_s also become tridiagonal matrices. Therefore, the SUSY-transformed CMT models with H_s maintain the dimension of an original CMT model, allowing only nearest-neighbor couplings[15-17]. Supplementary Figure 5 shows the variation of CMT parameters after the series of SUSY transformations of the binary photonic molecule in Supplementary Fig. 3.

In accordance with the varying amplitudes and frequencies of the potential shapes in the SUSY-transformed continuous potentials (in the main manuscript), the distributions of self-evolutions (Supplementary Fig. 5a) and interatomic couplings (Supplementary Fig. 5b) both become disordered after SUSY transformations. Note that ground-state annihilations are expressed in the form of the decoupling (black arrows)[15-17]. Decoupled atoms thus have self-evolution value lower than ρ_0 (Supplementary Fig. 5a).

Supplementary Figure 6 shows the eigenspectrum of each SUSY-transformed photonic molecule. Identical to the case of continuous potentials in the main manuscript, all of the spectral information in the original eigenspectrum (Supplementary Fig. 6a) are preserved during the series of SUSY transformations (Supplementary Figs 6b,6c), including the width and position of the bandgap and eigenbands. Note that the well-known cosine form of the eigenbands, which had been believed to originate from the *periodic* coupling[13], is also reproduced perfectly with disordered photonic molecules. Due to the ground-state annihilation, the lowest part of the SUSY-transformed eigenspectrum has eigenvectors localized to decoupled atoms (black arrows).

SUSY-based random-walk photonic molecules: real space design

To design the real structure corresponding to the CMT parameters used in Supplementary Fig. 5, the position and self-evolution term (a wavevector in a spatial CMT, and a resonant frequency in a temporal CMT) of each photonic atom should be determined. While the self-evolution can be easily manipulated through the design of photonic atoms, the coupling is mainly determined by the interatomic distance. The coupling coefficient is generally obtained as[4]

$$\kappa_{ij} = -\frac{i\omega}{4}\int \Delta\varepsilon \cdot \hat{e}_i \cdot \hat{e}_j^{*} ds\,, \tag{4}$$

where $\Delta\varepsilon$ is the perturbation of permittivity distribution and $\boldsymbol{e}_k$ is the normalized field pattern of the kth photonic atom. In the weak coupling regime, based on the evanescent field overlap, the coupling coefficient can be approximated as $\kappa_{ij} \sim c_1 \cdot exp(-c_2 \cdot \Delta x_{ij})$ where $c_{1,2}$ are platform-dependent constants and Δx_{ij} is the distance between the ith and j^{th} photonic atoms. Two unknown constants $c_{1,2}$ are determined when κ_{ij} for two different distances are defined, and then from $c_{1,2,}$ all of the coupling coefficients in Supplementary Fig. 5 can be converted to actual physical locations. The spatial distributions of the photonic molecules (obtained from Supplementary Fig. 5) are shown in Supplementary Fig. 7, presenting the spatially disordered

potential shape after the SUSY transformations.

From the results in Supplementary Fig. 7, we can now calculate the correlation of the potential shapes by applying the Hurst exponent[18,19]. Supplementary Figure 8 shows the Hurst exponents for the SUSY-transformed binary photonic molecule, as a function of the number of SUSY transformations. In agreement with the results in the main manuscript, although the original binary molecule has a strong negative correlation, the degree of long-range disorder in SUSY-transformed molecules increases rapidly with the series of SUSY transformations. The disorder comparable to the Brownian limit is also achieved, exhibiting a transition between negative and positive correlations.

In this Supplementary Note 2, we demonstrated the design of SUSY-based Bloch-like potentials in CMT-modelled discrete optical systems, perfectly preserving the width and position of bandgaps and the shape of each eigenbands. By employing the CMT implementation from the 1st-order approximation of Maxwell's equations, we showed that SUSY randomization of the potential can be applied to the system composed of generalized optical elements, transparent to polarizations and forms of eigenstates. Following this approach, the design of spatial or temporal Bloch-like wave devices with tunable correlations should be possible. For example, slow light propagation along disordered structures by SUSY-transforming the CROW, while preserving all of the spectral information, such as group velocity and its dispersion, can be envisaged. With the polarization-transparency of the CMT, the design of polarization-independent bandgaps can be expected as well, using dual-polarized optical elements[20]. The matrix-based SUSY randomization can also be extended into other basis systems allowing discretization, such as tight-binding analysis, plane-wave expansion methods, and density functional theory in quantum mechanics.

Supplementary Note 3. The annihilation of eigenstates from 2D SUSY transformations

Starting from the potential with the form $V_o(x,y) = V_{ox}(x) + V_{oy}(y)$, it can be shown that the eigenstates of the 2D potential are combinations of the eigenstates from the 1D potentials $V_{ox}(x)$ and $V_{oy}(y)$. By using the separation of variables for the 2D Schrodinger-like equation with an eigenstate $\psi(x,y) = \varphi(x)\cdot\phi(y)$,

$$\left[-\frac{1}{k_0^2}\cdot\frac{1}{\varphi}\cdot\frac{d^2\varphi(x)}{dx^2}+V_{ox}(x)\right]+\left[-\frac{1}{k_0^2}\cdot\frac{1}{\phi}\cdot\frac{d^2\phi(y)}{dy^2}+V_{oy}(y)\right]=\gamma, \quad (5)$$

each brace should be a constant, which is one of the eigenvalues of the 1D Schrodinger-like equation, with the potential $V_{ox}(x)$ or $V_{oy}(y)$ (green symbols in Supplementary Fig. 9).

Following the discussion in the Methods in the main manuscript, the annihilation by 2D SUSY transformations occurs not only in the ground state but also in all of the excited states that share a common 1D ground-state profile. The example of this phenomenon is shown in Supplementary Fig. 9 for the *x*-axis SUSY transformation.

Supplementary References

1. Schwartz, T., Bartal, G., Fishman, S. & Segev, M. Transport and Anderson localization in disordered two-dimensional photonic lattices. *Nature* **446**, 52-55 (2007).

2. Mukaiyama, T., Takeda, K., Miyazaki, H., Jimba, Y. & Kuwata-Gonokami, M. Tight-binding photonic molecule modes of resonant bispheres. *Phys. Rev. Lett.* **82**, 4623 (1999).

3. Peng, B., Özdemir, Ş. K., Zhu, J. & Yang, L. Photonic molecules formed by coupled hybrid resonators. *Opt. Lett.* **37**, 3435 (2012).

4. Haus, H. A. *Waves and fields in optoelectronics* (Prentice-Hall, 1984).

5. Bayindir, M., Temelkuran, B. & Ozbay, E. Tight-binding description of the coupled defect modes in three-dimensional photonic crystals. *Phys. Rev. Lett.* **84**, 2140 (2000).

6. Simovski, C. R. & Tretyakov, S. A. Local constitutive parameters of metamaterials from an effective-medium perspective. *Phys. Rev. B* **75**, 195111 (2007).

7. Caloz, C. & Itoh, T. *Electromagnetic Metamaterials: Transmission Line Theory and Microwave Applications* (John Wiley & Sons, 2005).

8. Longhi, S. Quantum-optical analogies using photonic structures. *Laser & Photon. Rev.* **3**, 243 (2009).

9. Yu, S., Piao, X., Koo, S., Shin, J. H., Lee, S. H., Min, B. & Park, N. Mode junction photonics with a symmetry-breaking arrangement of mode-orthogonal heterostructures. *Opt. Express* **19**, 25500 (2011).

10. Artar, A., Yanik, A. A. & Altug, H. Directional double Fano resonances in plasmonic hetero-oligomers. *Nano Lett.* **11**, 3694-3700 (2011).

11. Winick, K. A. Effective-index method and coupled-mode theory for almost-periodic waveguide gratings: a comparison. *Appl. Opt.* **31**, 757-764 (1992).

12. Peschel, U., Pertsch, T. & Lederer, F. Optical Bloch oscillations in waveguide arrays. *Opt. Lett.* **23**, 1701-1703 (1998).

13. Yariv, A., Xu, Y., Lee, R. K. & Scherer, A. Coupled-resonator optical waveguide: a proposal and analysis. *Opt. Lett.* **24**, 711-713 (1999).

14. Yu, S., Piao, X. & Park, N. Slow-Light Dispersion Properties of Multiatomic Multiband Coupled Resonator Optical Waveguides. *Phys. Rev. A* **85**, 023823 (2012).

15. Miri, M.-A., Heinrich, M., El-Ganainy, R. & Christodoulides, D. N. Supersymmetric optical structures. *Phys. Rev. Lett.* **110**, 233902 (2013).

16. Heinrich, M., Miri, M.-A., Stu¨tzer, S., El-Ganainy, R., Nolte, S., Szameit, A. & Christodoulides, D. N. Supersymmetric mode converters. *Nature Commun.* **5**, 3698 (2014).

17. Heinrich, M., Miri, M.-A., Stu¨tzer, S., Nolte, S., Christodoulides, D. N. & Szameit, A. Observation of supersymmetric scattering in photonic lattices. *Opt. Lett.* **39**, 6130-6133 (2014).

18. Hurst, H. E. Long-term storage capacity of reservoirs. *Trans. Amer. Soc. Civil Eng.* **116**, 770-808 (1951).

19. Roche, S., Bicout, D., Maciá, E. & Kats, E. Long range correlations in DNA: scaling properties and charge transfer efficiency. *Phys. Rev. Lett.* **91**, 228101 (2003).

20. Zhang, Y., McCutcheon, M. W., Burgess, I. B. & Loncar, M. Ultra-high-Q TE/TM dual-polarized photonic crystal nanocavities. *Opt. Lett.* **34**, 2694-2696 (2009).
